# Salinity Stress Influences the Main Biochemical Parameters of *Nepeta racemosa* Lam.

**DOI:** 10.3390/plants12030583

**Published:** 2023-01-29

**Authors:** Constantin Lungoci, Iuliana Motrescu, Feodor Filipov, Cristina Mihaela Rimbu, Carmenica Doina Jitareanu, Carmen Simona Ghitau, Ioan Puiu, Teodor Robu

**Affiliations:** 1 Department of Plant Science, Faculty of Agriculture, Iasi University of Life Sciences, 3 Sadoveanu Alley, 700490 Iasi, Romania; 2 Department of Exact Sciences, Faculty of Horticulture, Iasi University of Life Sciences, 3 Sadoveanu Alley, 700490 Iasi, Romania; 3 Research Institute for Agriculture and Environment, Iasi University of Life Sciences, 14 Sadoveanu Alley, 700490 Iasi, Romania; 4 Department of Pedotechnics, Faculty of Agriculture, Iasi University of Life Sciences, 3 Sadoveanu Alley, 700490 Iasi, Romania; 5 Department of Public Health, Iasi University of Life Sciences, 8 Sadoveanu Alley, 707027 Iasi, Romania

**Keywords:** salt stress, *Nepeta*, soil salinity, salt tolerance, bioactive compounds

## Abstract

In this work, the effects of salt stress on *Nepeta racemosa* Lam. were studied to analyze the possibility of using it as a potential culture for salinity-affected soils. A total of nine concentrations of salts—NaCl (18, 39, and 60 mg/100 g soil), Na_2_SO_4_ (50, 85, and 120 mg/100 g soil), and a mixture (9 g NaCl + 25 g Na_2_SO_4_, 19 g NaCl + 43 g Na_2_SO_4_, and 30 g NaCl + 60 g Na_2_SO_4_/100 g soil)—simulated real salinity conditions. Environmental electron microscopy offered information about the size and distribution of glandular trichomes, which are very important structures that contain bioactive compounds. The chlorophyll pigments, polyphenols, flavonoids, and antioxidant activity were determined based on spectrophotometric protocols. The results have shown a different impact of salinity depending on the salt type, with an increase in bioactive compound concentrations in some cases. The highest polyphenol concentrations were obtained for Na_2_SO_4_ variants (47.05 and 46.48 mg GA/g dw for the highest salt concentration in the first and second year, respectively), while the highest flavonoid content was found for the salt mixtures (42.77 and 39.89 mg QE/g dw for the highest concentrations of salt in the first and, respectively, the second year), approximately 100% higher than control. From the Pearson analysis, strong correlations were found between chlorophyll pigments (up to 0.93), antioxidant activity and yield for the first harvest (up to 0.38), and antioxidant activity and flavonoid content for the second harvest (up to 0.95). The results indicate the possibility of growing the studied plants in salt-stress soils, obtaining higher concentrations of bioactive compounds.

## 1. Introduction

Stress factors are an important issue in agriculture, causing huge losses estimated at more than 50% [1]. In agriculture, abiotic stress factors can be extreme temperatures, extreme precipitation conditions (floods or drought), radiation, chemical stressors (deficiencies or excesses of nutrients, soluble salts, alkalinity, contaminants, etc.), soil physical factors, and also biological [2,3]. Salinity is among these abiotic stressors causing important economic losses and endangering food global security [4]. This is common in arid and semi-arid areas, during long drought periods, but it is also affecting approximately 20% of irrigated fields [5,6]. According to the FAO, approximately 7% of global agricultural lands are affected by salinity, and the percentage is continuously increasing, especially due to climate change [7]. In Europe, the surface of salt-affected soils is approximately 3.8 billion ha, among which 500 thousand ha are in Romania [8]. High salinity is mostly due to high concentrations of Na^+^ and Cl^−^ in the soil that produce hyperosmotic and hypertonic solutions that stop water and nutrient uptake by plants [9]. Salinity stress at the plant level perturbs biochemical and physiological processes in the cell or even at the whole-plant level, and can be ionic and/or osmotic stress [10,11]. The action path can have an immediate effect, impacting the rhizosphere and some exposed parts of the plants and long-term effects on the whole plant depending on the stress [12]. At the cell level, it causes metabolic toxicity, affects photosynthesis, leads to the production of oxygen reactive species (ROS), and causes membrane disruption, and even programmed cell death [9,10,11,12,13]. At the plant level, the effects become visible in time (after days up to months) because of the limited capacity of the cells to accumulate ions [14]. Some physiological changes are also found such as severe necrotic lesions on the leaves, reduced growth, a reduced number of leaves, and affected plant reproduction [1,5,9,12,13]. There are several ways to mitigate the effect of different abiotic stresses such as enzymes and non-enzymatic antioxidants [15,16,17].

In medicinal plants, salt stress has been shown to produce different effects depending on the analyzed species and salt stress level. In the case of *H. cannabinus*, salt stress enhanced the total antioxidant capacity, stimulating the production of some bioactive compounds but only for low-salinity conditions, and some compounds were not synthesized anymore for high salt stress, although new compounds were detected [18]. Similar results of stimulation under low-salt stress conditions were obtained in the case of *Glycyrrhiza uralensis* [19], while researchers found a strong dependence of the concentration of the bioactive compounds on the salinity for *Lonicera japonica* [20].

*Nepeta* sp. belongs to the *Laminaceae* family and has approximately 300 species with high phenotypic plasticity and it originates from South-West Asia [21,22,23]. *Nepeta racemosa* Lam. is characterized by a high content of nepetalactone, which are secondary metabolites with a wide range of uses [21,22,23,24]. The beneficial effects can be divided into several categories depending on the bioactivity: the antiseptic, astringent, and antiasthma properties are used in medicine, the antibacterial and preservative properties are put to use in the food industry, the bio-herbicidal and pheromonal properties for aphides abilities are used in plant protection [21,22,23,24,25,26,27].

Because it is predicted that by 2050 approximately half of agricultural soils will be affected by salinity, it is crucial to find some strategies to stimulate and increase crop production efficiency and find useful plants that can adjust and even thrive in these conditions [3,28]. The full range of effects induced by salt stress in Nepeta is unclear. Despite having a large range of uses, the species is not extensively studied. Some researchers found that salt stress significantly reduces the germination and seeding growth in *Nepeta persica* exposed under laboratory conditions [29,30]. In our previous study, we found that salt stress can indeed lead to negative effects on *Nepeta cataria* L such as a decrease in yield and concentrations of photosynthetic pigments, but can also stimulate the production of bioactive compounds and antioxidant activity in some conditions; overall, the species react well to high salinity levels [21]. In this work, we aimed to test the influence of sodium chloride (NaCl) and sodium sulfate (Na_2_SO_4_), the two most common salts in Romanian soils affected by salinity, on *Nepeta racemosa* Lam. to increase the economic value of these salinity-affected soils and capitalize the potential of the species. Thus, we focused on analyzing the effects of salt stress on growth and some biochemical compounds with important bioactive properties.

## 2. Results

### 2.1. Biomass Yield

**Figure 1:**
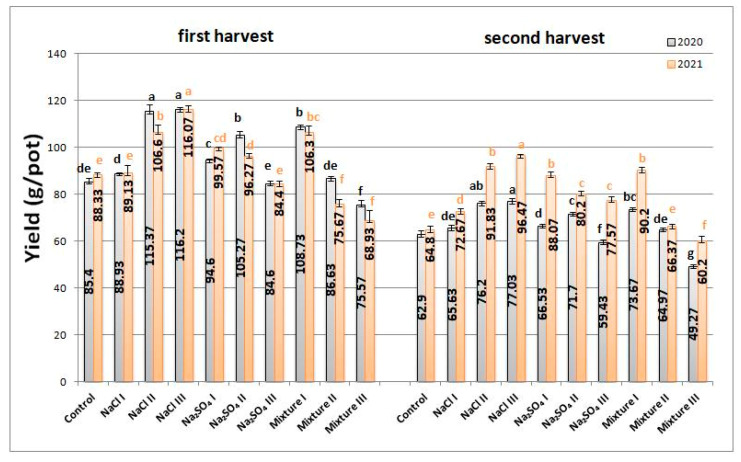
Average yield per vegetation pot for *Nepeta racemosa* Lam. for both analyzed years with significant differences at 0.05 level indicated with letters.

Figure 1 shows that some salt concentrations stimulated the biomass yield. In 2020, the highest biomass values were obtained for NaCl III (116 ± 0.76 g/pot) with an increase of 36.07% compared to control—NaCl II (115 ± 3 g/pot), mixt. I (108.73 ± 0.73 g/pot), Na_2_SO_4_ II (105.27 ± 1.57 g/pot). A lower biomass than the control indicating inhibition of biomass was determined for a high concentration of Na_2_SO_4_ and a mixture variant, meaning Na_2_SO_4_ III (84.6 ± 1.18 g/pot) and mixt. III (75.57 ± 1.81 g/pot), respectively. The maximum decrease measured was 11.52% for mixt. III in the second harvest.

For the second year, 2021, the values were similar to the first, 2020. The most noticeable changes were detected for the second harvest, where the production was higher than that of the previous year, as can be seen in Figure 1. Analyzing both years, we can say that NaCl stimulated green mass production, while for Na_2_SO_4_, only low concentrations (50 mg/100 g soil) had a similar effect. The highest concentration of salt mixture inhibited growth.

### 2.2. Morphological Modifications

**Figure 2:**
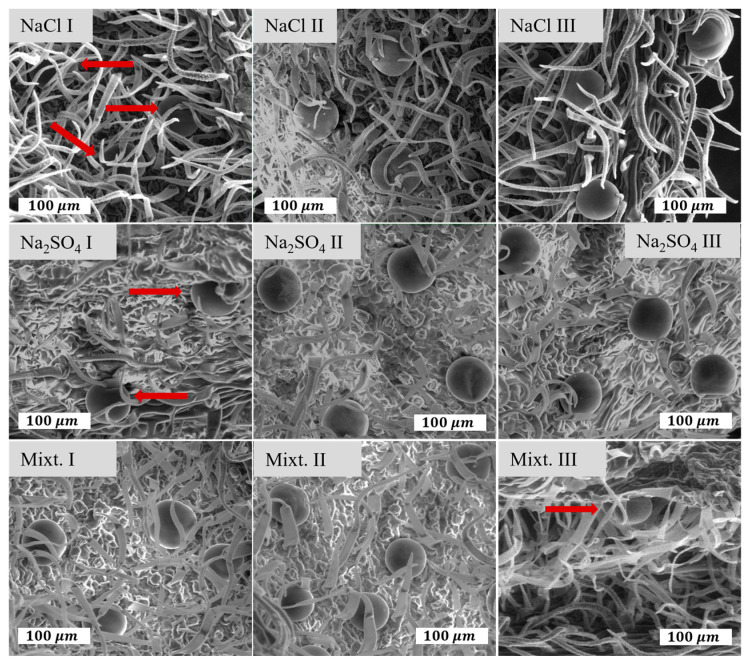
ESEM microimaging of the surface of leaves with the identification of the granular trichomes for all variants.

**Figure 3:**
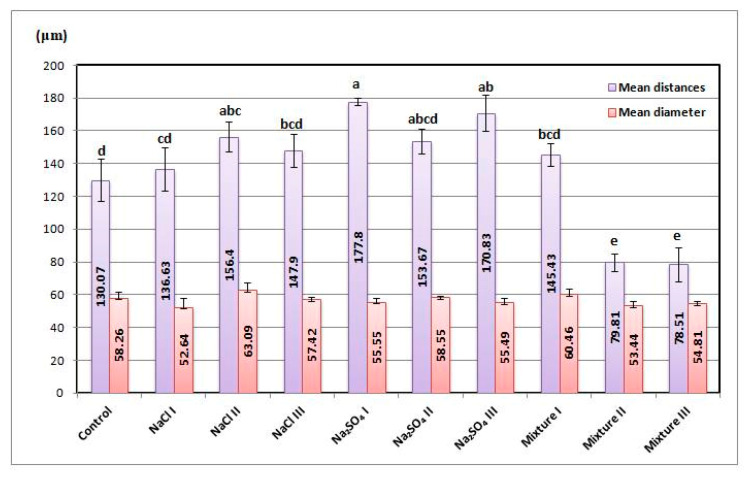
Information related to the granular trichomes: mean diameters and mean distance in between trichomes, respectively, with significant differences at 0.05 level indicated with letters.

Glandular trichomes are the main secretory structures for *Nepeta racemosa*. ESEM images are presented in Figure 2, while the information related to the average size of the trichomes and distribution measured from the ESEM data is shown in Figure 3. The size of the trichomes changed in a narrow range: 52.64–60.46 μm. The highest values correspond to NaCl II and mixt. I variants. On the other hand, it is clearly seen that the density of trichomes changed significantly with large variations from one variant to another. The larger average distance between trichomes was determined for Na_2_SO_4_ I (177.8 ± 19.6 μm), while the shortest was measured for mixt. III (78.51 ± 5.21 μm). Large distances between trichomes can be seen as low surface density and short average distances as high density.

### 2.3. Biochemical Analyses

**Figure 4:**
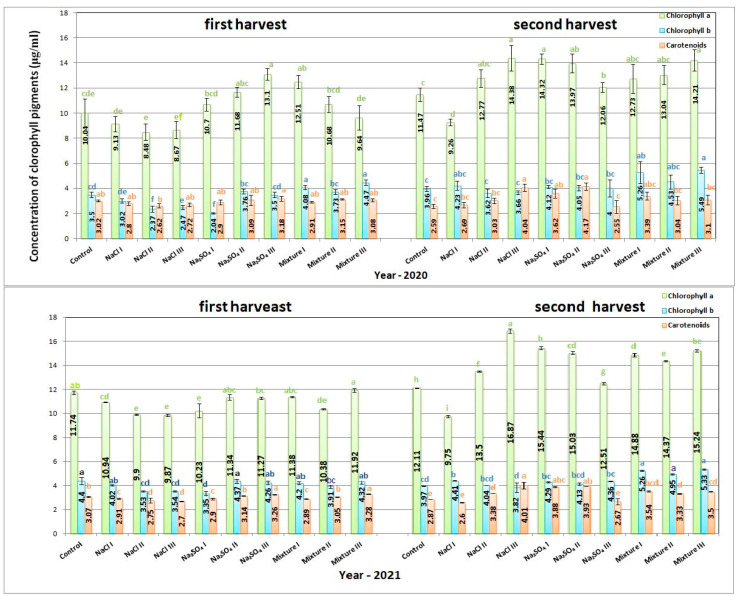
The concentrations of photosynthetic pigments for *Nepeta racemosa* Lam. in 2020 and 2021, with significant differences at 0.05 level indicated with letters.

In 2020, the highest chlorophyll a content was measured for Na_2_SO_4_ III, with a value of 13.1 ± 0.44 μg/mL for the first harvest and 14.38 ± 1.03 μg/mL for NaCl III in the second harvest, respectively, and an increase of approximately 30% as compared to control crops. Chlorophyll b had maximum values in the first harvest for mixt. III variant, with 4.47 ± 0.22 μg/mL, meaning an increase of 23.71% compared to the control plants, while for the second harvest, the same variant measured the maximum concentration with an increase of 38.63%. The highest carotenoid content was recorded for Na_2_SO_4_ III (3.18 ± 0.19 μg/mL) for the first harvest and Na_2_SO_4_ II (4.17 ± 0.3 μg/mL) for the second, with an increase of approximately 60% compared to the control. All the values of the pigments measured for all variants in 2020 and 2021 are shown in Figure 4 The values in 2021 had a similar trend to those in 2020. The highest chlorophyll a content was measured for the second harvest of the NaCl III variant, with an increase of 39.3% compared to the control. In the first harvest, the maximum value was found for mixt. III, with just a small increase compared to the control of approximately 1.59%. Similar to 2020, the highest chlorophyll b concentration was obtained for mixt. III variant with a 34.25% increase compared to the control for the second harvest, while for the first, all values were lower than for the control variant. The carotenoid content was maximum for mixt. III, with only a small increase of 6.84% for the first harvest. For the second harvest, NaCl III exhibited an increase of almost 40% compared to the concentration of the control variant.

**Figure 5:**
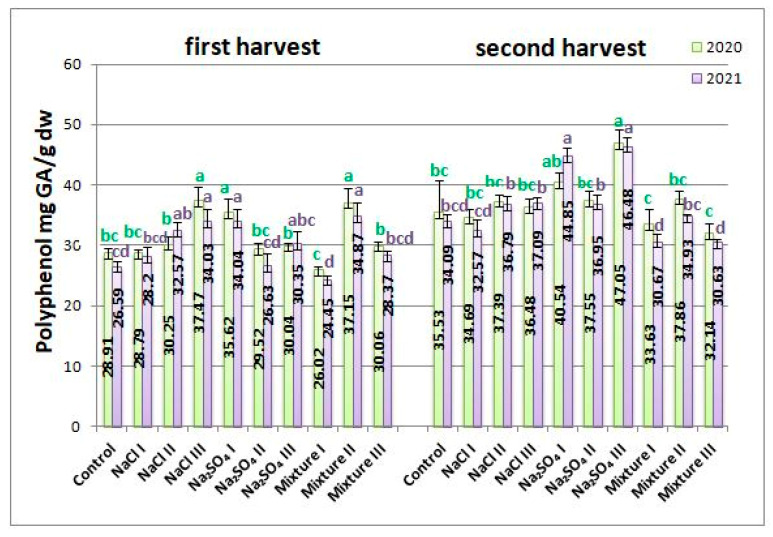
Average polyphenol content in *Nepeta racemosa* Lam. variants from 2020 and 2021, with significant differences at 0.05 level indicated with letters.

The polyphenols in *Nepeta racemosa* Lam. extracts give some of its antioxidant and antimicrobial properties. The analysis of the polyphenol content with the Folin–Ciocalteu method indicated that in 2020, it was higher for the second harvest, as compared with the first. The maximum value in the first harvest was measured for NaCl III (37.47 ± 1.23 mg GA/g dw), while the minimum value was measured for mixt. I (26.02 ± 0.52 mg GA/g dw), which was also lower than the control, as can be seen from the data presented in Figure 5. For the second harvest of 2020, the highest value of polyphenols was detected in the case of the Na_2_SO_4_ III variant (47.05 ± 1.48 mg GA/g dw), 32.42% higher than the control. The minimum concentration, which was also lower than the control, was measured for the mixt. variants I and III, 10% lower than the control (Figure 5).

In 2021, the trend was similar to 2020, with small exceptions. At the first harvest, the highest concentrations were measured for mixt. II, Na_2_SO_4_ I, and NaCl III, with an increase of up to 28% compared to the control. The minimum concentration was found just as for 2020 for mixt. I variant (24.45 ± 0.35 mg GA/g dw), lower by a little more than 15% than the control. In the second harvest, Na_2_SO_4_ III exhibited the highest amount of polyphenols, with an increase of 30.82% compared to the control. Mixt. I and mixt. III gave decreased concentrations compared to the control, by up to 10% (Figure 5).

**Figure 6:**
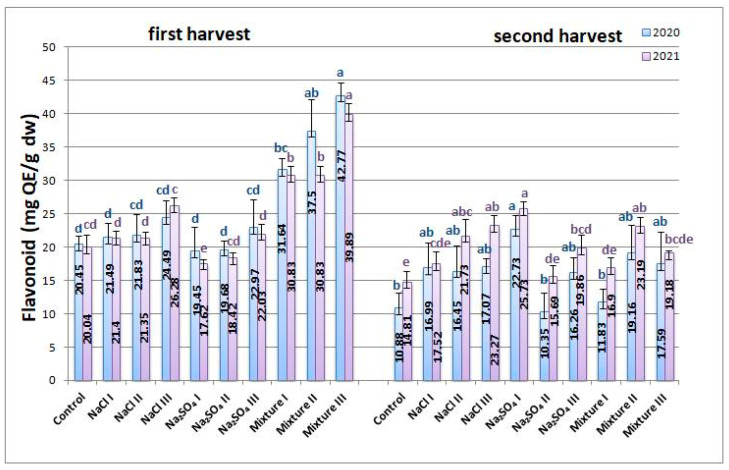
Average flavonoid content from *Nepeta racemosa* Lam. in 2020 and 2021, with significant differences at 0.05 level indicated with letters.

The flavonoid concentrations increased more for the first harvest than for the second for both years, as can be seen in Figure 6. The highest increase was found for mixt. variants in the first harvest, up to more than 100% in some cases, while for the other conditions, the increase was much lower, up to 20% for 2020 and approximately 30% for 2021. For the second harvest, the flavonoid concentration was higher than for the control in all variants in both years.

**Figure 7:**
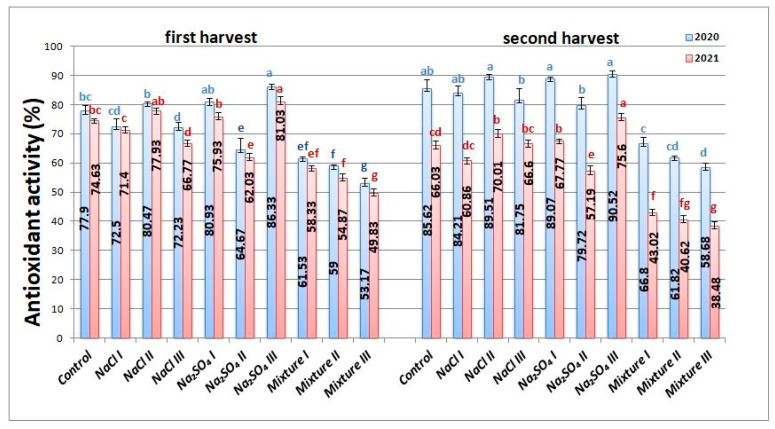
Antioxidant activity of *Nepeta racemosa* Lam. variants in 2020 and 2021, with significant differences at 0.05 level indicated with letters.

The antioxidant activity slightly increased for a few of the variants. For the first year, the maximum values were measured for Na_2_SO_4_ III both in the first harvest (86.33 ± 0.81%) and second harvest (90.52 ± 1.06%), while mixt. III exhibited the lowest values in both harvests, with a serious decrease of approximately 31% for both. The data are presented in Figure 7. In 2021 (Figure 7), most variants had the same behavior related to the antioxidant activity as in the previous year, with a much-pronounced decrease in the case of mixt. III variant of approximately 33% in the first harvest, and 41% in the second harvest.

**Figure 8:**
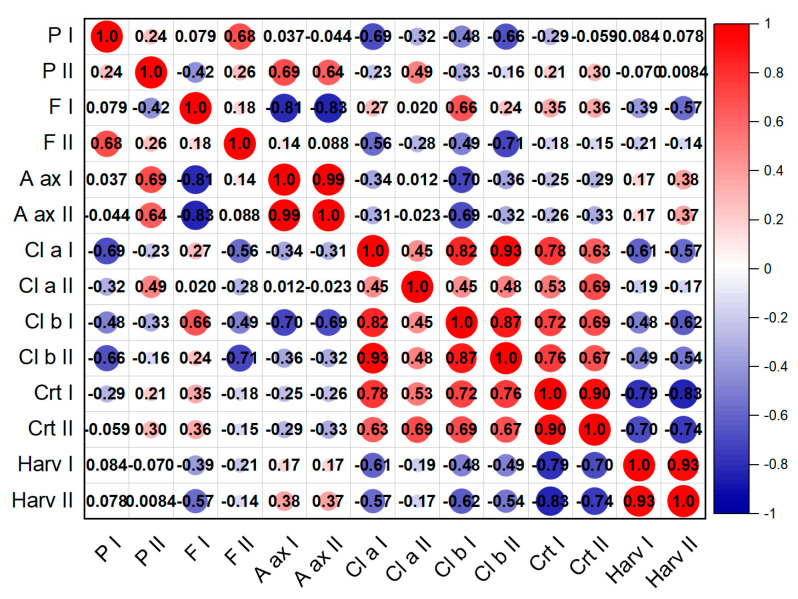
Pearson correlation matrix for the first harvest in 2020 and 2021 in *Nepeta racemosa* Lam. variants.

**Figure 9:**
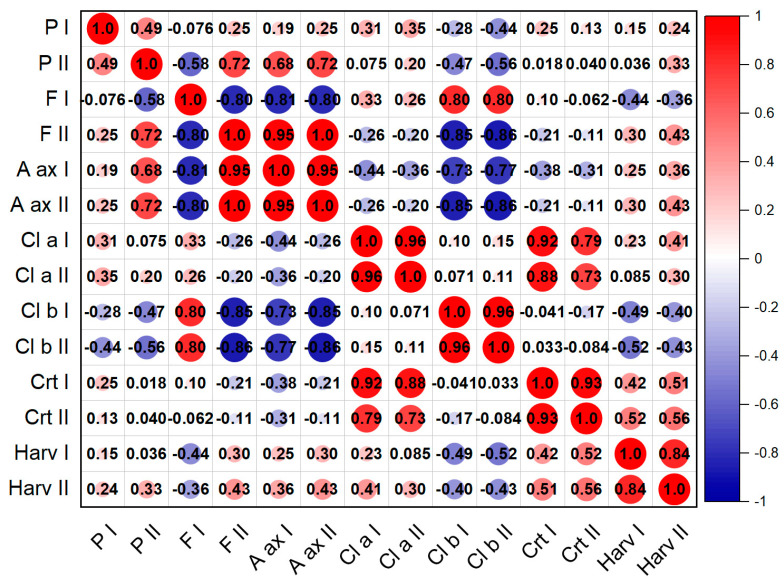
Pearson correlation matrix for the second harvest in 2020 and 2021 in *Nepeta racemosa* Lam. variants.

The Pearson correlation analysis in the studied years corresponding with the studied variants is presented in Figure 8 and Figure 9, for the first and second harvest, respectively. In the case of the first harvest, we can see a strong correlation in the level of chlorophyll pigment accumulation for the two years (R^2^ = 0.90), antioxidant activity (R^2^ = 0.99) but also green mass accumulation (R^2^ = 0.93). Negative correlations were found in the production of flavonoids (R^2^ = −0.81) and antioxidant activity (R^2^ = −0.83), with flavonoids presenting a negative correlation with most studied parameters. For the second harvest, we found positive correlations between the antioxidant activity and flavonoid content for both years (R^2^ = 0.95) and also chlorophyll pigments (R^2^ = 0.88). Strongly negative correlations were found in the relationship between the chlorophyll pigments, and flavonoid and antioxidant activity.

## 3. Discussion

The biomass analysis indicated that the NaCl concentrations used in this study had a beneficial effect on the plants, stimulating biomass production, while for Na_2_SO_4_ and mixed salts-treated variants, an inhibitory effect was evidenced as the salt concentration increased. These results could be attributed to the osmotic stress that interferes with the metabolic processes, reducing the energy available for growth and development [31,32]. Similar behavior in the case of NaCl was reported by Neffati et al. for *Coriandrum sativum* and concentrations of 50 and 75 mM [32], while others reported a strong increase in the biomass for the same concentrations for *Salvia officinalis* L. [33]. The different behavior could be attributed to the difference between species, some responding similarly to the same stress factors, some having opposite effects.

Glandular trichomes are the main secretory structures for *Nepeta racemosa* Lam. In normal growing conditions, they have a diameter of approximately 50 μm [34] and contain approximately 30 ng of essential oil [35]. Abiotic stress factors strongly influence the glandular trichomes, affecting their size and number [36]. In the case of the trichomes diameters, despite some changes noticed, there was no statistical significance to clearly indicate an effect. However, under salinity stress, it seems that the surface density of the trichomes was decreasing, similar to what we had measured using electron microscopy as we presented in Figure 3. The values of the distances between trichomes we found were higher than those reported in the literature [34,37] for most of the cases, except in the case of high concentrations of the salt mixture, where the mean distances between trichomes decreased so that their density increased.

Abiotic stress factors can influence photosynthetic efficiency, thus determining the concentrations of chlorophyll pigments is very important to establish the tolerance of the plants to different stressors. Recent studies show that salinity stress leads to an increased number of chloroplasts [38]. Our results have shown that high salt concentrations lead to high carotenoid concentrations. Similar results have been reported by other researchers, for example in the case of *Fagopirum esculentum* M., for which a concentration of 100 mM NaCl led to an increase of 40% of carotenoid content [39]. The reported results are variable between species and treatments. For low salt concentrations, *Portulaca oleracea* L. exhibited an increased concentration of both chlorophyll a and b, while for high salt concentrations, both chlorophyll pigment concentrations decreased [40,41]. Our results presented in Figure 4 indicate a stimulating effect of up to 30% for the salt concentrations used in this study, especially in the case of the Na_2_SO_4_ III variant. The differences between the harvests are mostly due to different climate conditions during plant development.

The phenolic compounds influence seed germination, the growth of the plants, biomass accumulation, and metabolism [42]. There is proof that high concentrations of phenols are found when the plant is under stress [43], with secondary metabolites being produced by the plant to counteract the stressors, adjust to the conditions, and survive [42]. *Aegiceras corniculatumpun* exhibited an increased concentration of polyphenols after growing under salinity stress for a concentration of 250 mM NaCl [44]. The same stimulating behavior was also reported for wheat [45], mint [46], salvia [47], echinacea [48], and others, believed to be a counteracting effect to salinity stress. This also corresponds with the moderate correlation found between the concentrations of phenolic compounds and photosynthetic compounds, as well as between their concentration and the antioxidant activity evaluated in our study.

Similar to the phenolic compounds, flavonoid production was stimulated as a result of salinity stress. Our results indicated that the highest concentrations were found for the variants with the highest salt concentrations. This also correlated well (0.68) with polyphenol concentrations. Similar behavior has been reported for other plant species such as *Cynara scolymus* L. [49], and *Verbena officinallis* L. [50]. A concentration of 100 mM NaCl combined with the use of *Ascophyllum nodosum* led to an increased flavonoid concentration in *Vigna radiata* L. [51], while 150 mM had the same stimulating effect for *Trigonella foenum-graecum* L. [52], confirming the results presented in this study.

The antioxidant activity is given by all the reactions between the most important biochemical components of the plant to neutralize the reactive oxygen species (ROS) produced as a response to some stress factor such as salinity [53]. ROS will act as signaling molecules, opening signaling pathways as a response. They also produce irreversible damage at the cell level due to their highly oxidative properties, leading to morphological changes and increasing plant resistance [54,55]. Lipid peroxidation and oxidative stress were evidenced by the increase in malondialdehyde (MDA) in *Rosa damascena* under salt stress conditions, with the same report indicating a reduction in antioxidant activity [56]. A similar increase in MDA has been reported in other works as well for salt concentrations of up to 100 mM [57]. Studies performed on *Mentha pulegium* confirmed that a concentration of 100 mM NaCl has a stimulating effect on second metabolite production, and thus antioxidant activity [58]. Another study has shown that for a concentration of 50 mM NaCl, the antioxidant activity of *Mentha spicata* increased, as evaluated using FRAP, ABTS, and DPPH [59].

## 4. Materials and Methods

### 4.1. Experimental Design

**Figure 10:**
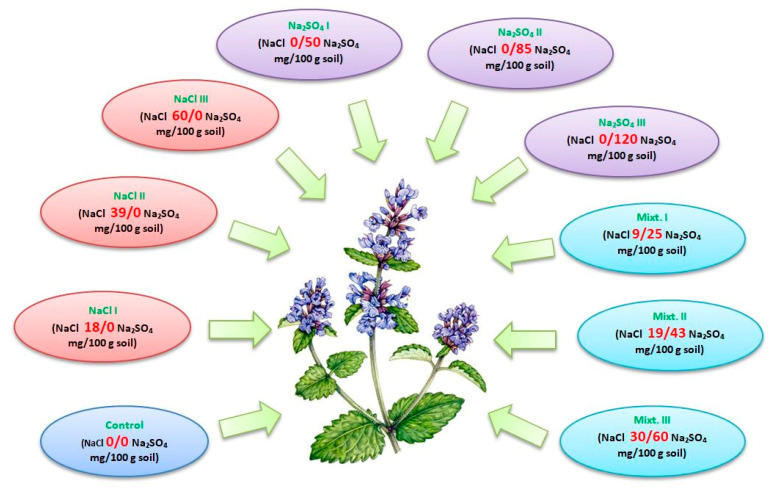
Experimental design scheme.

The experiments were performed in a completely random design (CRD) and were carried out for two years (2020 and 2021) in the research field for medicinal, aromatic and spice plants from the Faculty of Agriculture, Iasi University of Life Sciences, Romania. The Mitscherlich vegetation pots, with a volume of 10 dm^3^ each, were filled with homogenized soil, 7 kg each, calculated at the level of field water capacity. The salinity levels were established according to the pedologic classification of the soils in the Moldavian area. The soil was obtained from the horizon corresponding to 0–19 cm depth, and then vegetal residues and other big impurities were removed. The soil chemical analysis indicated the following parameters for the soil: pH—7.9, phosphorus—31 ppm, potassium—229 ppm, humus—3.49%, calcium carbonate—4.77%, total nitrogen—0.176%, organic carbon—2.02%, carbon to nitrogen ratio—9.84, and the total content of soluble salts in soil solution—53 mg/100 g soil. The seeds were sown at the end of March and, by the end of April, the seedlings were placed in the vegetation pots on a brown peat layer (0–6 mm size), with pH 5.5–5.9 and supplemented with NPK fertilizer (3.28 g N_16_P_16_K_16_ per pot). Nine variants with four repetitions were tested with different salt concentrations as indicated in Figure 10, as well as untreated plants were used as control samples. During the vegetation, the humidity of the soil was kept at the level of water field capacity; the extra water remaining from the Mitscherlich pots was collected and used for the same conditions. The top layer of soil was covered in the mulch. The cultures flower twice a year, and these are considered harvest times. For the biomass yield, the whole leave mass on each variant was measured. For the other analyses, upper leaves from the main stem of the plants were collected.

### 4.2. Climate Conditions

Climate changes have been affecting the average temperature and precipitation in recent years. The highest difference recorded during the experiments was in March and October 2020, at +3, and +2 °C, respectively. The second year of the experiment, 2021, was cooler, with the average temperature being lower than normal by approximately 1–2 °C.

The amplitude of precipitation was very high. The driest months were September 2020 and October 2021. Much higher precipitation than the multiannual average was recorded in August and October 2020. In 2021, the average quantity was close to the multiannual value for all the studied periods. The data were collected from the “Vasile Adamachi” meteorological station in Iasi, Romania (47.195925, 27.552277) and are presented in Table 1.

### 4.3. Morphological Analysis

Environmental Scanning Electron Microscopy (ESEM) was used to image the surface of the leaves, and identify and quantify the glandular trichomes (Quanta 450, FEI, Thermo Fisher Scientific, Hillsboro, OR, USA). The samples did not need any special preparation before imaging since the ESEM works in the environmental mode, so they were analyzed as they are. Small pieces of the leaves (5 × 5 mm) from the upper part of the plants were cut and carefully placed on top of aluminum stubs, glued with double-side carbon tape, with the abaxial surface exposed. The measurements were performed at a working pressure of 70 Pa in a low-vacuum mode, using an electron beam accelerated at 15 kV and a spot size of 2, for a working distance of 10 mm.

### 4.4. Biochemical Analyses

The chlorophyll pigments were determined from fresh leaves (0.5 g) after extraction in 80% acetone. Spectrophotometric measurements were performed with a UV–Vis spectrophotometer (SP-V1100, DLAB Scientific Co., Ltd., Beijing, China) at 663.2, 646.8, and 470 nm, and the values (in μg/mL) were calculated according to Lichtenthaler formulas adapted by Wellburn [60,61]:

(1) $$C_{a}=12.25\cdot A_{663.2}-2.79\cdot A_{646.8}$$

(2) $$C_{b}=21.50\cdot A_{646.8}-5.10\cdot A_{663.2}$$

(3) $$C_{x+c}=\frac{1000\cdot A_{470}-1.82\cdot C_{a}-85.02\cdot C_{b}}{198}$$

The phenol content, flavonoid content, and antioxidant activity were determined using spectrophotometric protocols from plant extract. An amount of 0.5 g of green mass (freshly collected at the same level for representative plants for each variant) in 95% ethylic alcohol was sonicated for 15 min and then incubated for 24 h at 4 °C. The filtered extract was used for analyses [62]. The phenol content was determined with the method based on the Folin–Ciocalteu reactive, the absorbance at 760 nm serving for calculating the concentrations expressed in GA/g dw after a calibration curve obtained for gallic acid (r^2^ = 0.9983) [63]. The flavonoids were determined by mixing 0.25 mL plant extract with 5% NaNO_2_ and 10% AlCl_3_ at basic pH by adding NaOH 1 M. The readings were taken at 510 nm, then the content was determined based on a calibration curve obtained for different concentrations of quercetin (r^2^ = 0.9912) [64,65,66]. The antioxidant activity was determined based on the reduction of 2,2-diphenyl-1-picrylhydrazyl (DPPH), the reaction with the extract changing the color from violet to yellow. The spectrophotometric measurements at 510 nm were used to determine the antioxidant activity expressed in percentage (% inhibition) relative to the maximum absorbance of DPPH solution as follows:

(4) $$DPPHsc\%=\frac{{\left(Abs\;control\right)}_{t=x\;min}-{\left(Abs\;sample\right)}_{t=x\;min}}{{\left(Abs\;control\right)}_{t=x\;min}}\times 100$$

where Abs control means the absorbance of the DPPH solution without the extract, while Abs sample is the absorbance of DPPH combined with the sample extract [67,68,69].

### 4.5. Statistical Analysis

One-way analysis of variance test (ANOVA) (IBM SPSS v14) was used to determine the significant differences between the variants; Tukey’s multiple comparison test was employed when the results were significant (*p* < 0.05). The results are presented as average values with standard deviations. To find correlations between the studied parameters, the Pearson correlation test was performed using Origin Pro.

## 5. Conclusions

Salinity stress on all analyzed conditions had a strong impact on *Nepeta racemosa* Lam., the results being in very good agreement with others reported in the literature for other aromatic plants and our previous study on *Nepeta cataria* L. as well. While we found stimulation of biomass production for NaCl variants, there was a decreasing trend of the biomass with increasing salt concentration for Na_2_SO_4_ and mixture variants, showing the possible different pathways initiated by Cl^−^ and SO_4_^2−^.

The most affected by the salinity seemed to be the variants where the mixture of salts was applied, with decreased green mass, trichomes densities, and antioxidant activity, but high levels of bioactive compounds. NaCl seemed to have a milder influence on *Nepeta racemosa* Lam., with increased yield, polyphenol and flavonoid contents, while Na_2_SO_4_ exhibited yield values comparable to control, but had increased concentrations of photosynthetic pigments, and decreasing polyphenol and flavonoid contents. The antioxidant activity and increased chlorophyll contents could also be connected with the increased temperature above average in the second harvest, strongly correlated to the polyphenol content and biomass production.

We can conclude that, in some conditions, salinity stress can trigger the increase in bioactive compounds and antioxidant activity in *Nepeta racemosa* Lam., but in the conditions simulating Moldavian soil salinity (corresponding with mixt variants), it is difficult to say that there are huge advantages for cultivating this species for the extraction and use of the bioactive compounds. However, this information could be useful for other areas where salinity corresponds with the salt concentrations at which the production of the bioactive compounds was stimulated. More investigations need to be performed to elucidate the impact of salinity and establish the economic reliability of *Nepeta racemosa* Lam. cultures and uncover other potential aromatic plant species for this purpose.

## Figures and Tables

**Table 1:** Mean monthly values of precipitations and temperatures at the experimental site.

**Average temperature (°C)**								
	**March**	**April**	**May**	**June**	**July**	**August**	**September**	**October**
**2020**	**7.34**	**11.01**	**14.29**	**20.97**	**22.42**	**23.27**	**19.2**	**13.79**
**2021**	**3.7**	**8.3**	**15.2**	**19.8**	**23.2**	**20.9**	**14.6**	**8.51**
**Average precipitation (mm)**								
**2020**	**65.4**	**56.4**	**87**	**115**	**71.6**	**155.4**	**12.4**	**72.8**
**2021**	**29.2**	**44.8**	**55.8**	**83.7**	**71.3**	**57.4**	**47**	**10.2**

## Data Availability

Not applicable.
